# Neurodegeneration of the cornea and retina in patients with type 1 diabetes without clinical evidence of diabetic retinopathy

**DOI:** 10.3389/fendo.2022.790255

**Published:** 2022-10-05

**Authors:** Josie Carmichael, Hassan Fadavi, Mitra Tavakoli

**Affiliations:** ^1^ Exeter Centre of Excellence for Diabetes Research, National Institute for Health and Care Research (NIHR) Exeter Clinical Research Facility, University of Exeter Medical School, Exeter, United Kingdom; ^2^ Peripheral Neuropathy Group, Imperial College, London, United Kingdom

**Keywords:** neurodegenaration, type 1 diabetes mellitus, retinopathy, neuropathy, corneal confocal microscopy, optical coherance tomography

## Abstract

**Aim:**

Diabetic retinopathy (DR) is widely considered the earliest and most common microvascular complication of diabetes. However, recent studies have shown that retinal nerve fiber layer and corneal nerve abnormalities may be present in diabetic patients without retinopathy. This preliminary study aimed to establish if structural and functional changes in the nerve fiber layer of the retina and cornea occur in patients with type 1 diabetes (T1DM) without retinopathy.

**Methods:**

Twenty patients with T1DM, without clinical evidence of retinopathy (Age: 47.0 ± 2.5 years; Duration diabetes: 27.0 ± 3 years) and 15 age-matched healthy control subjects underwent detailed medical neurological examinations. Ophthalmic examinations using Spectral Domain Optical coherence tomography (SD-OCT), Standard Automated Perimetry (SAP), Flicker Defined Form High Edge Perimetry (FDF), Corneal Confocal Microscopy (CCM) and Non-contact corneal Aesthesiometry (NCCA) were performed to quantify the structure and function of the nerves in the retina and cornea, respectively.

**Results:**

At the structural level, retinal nerve fiber layer thickness (RNFL) was significantly reduced in the superior nasal (p=0.001) and inferior temporal (p=0.004) sectors, in diabetic patients. Retinal ganglion layer function was reduced in the patient group when assessed using Flicker Defined Form Perimetry (FDF), but this was not significant. The function of the cornea assessed by corneal sensitivity, using a non-contact corneal aesthesiometer (NCCA), was significantly reduced (p=0.001). Structural assessment of corneal nerves using corneal confocal microscopy (CCM) showed reduction at corneal nerve fiber density (CNFD) (p=0.01), branch density (CNBD) (p=0.006) and length (CNFL) (p=0.01) in patients with diabetes. Compared to control subjects, the percentage of abnormality in patients with T1DM for RNFL was 32% while the FDF was abnormal in 61% of patients. Corneal abnormality was observed in 47% for NCCA, 28% for CNFD, and 17% for CNFL. There was no correlation between neuronal damage in the retina and cornea.

**Conclusions:**

Neuronal abnormalities were observed in both the retina and cornea of diabetic patients without evidence of retinopathy. The prevalence of structural and functional changes was higher in the retina compared to the cornea. This preliminary study suggests that structural neuronal changes may occur in parallel and correlate with functional changes. The assessment of corneal and retinal nerve structure may be clinically useful for detecting and monitoring the earliest stages of diabetic microvascular abnormalities.

## Introduction

Diabetic retinopathy (DR) is widely considered one of the earliest and most common microvascular complications of diabetes (1, 2). Although microvascular damage is the classic hallmark of DR, there is emerging evidence to suggest that retinal neurodegeneration may precede retinopathy (3). Indeed, studies have shown that hyperglycemia affects the neural layers of the retina, leading to visual symptoms such as reduced contrast sensitivity, abnormal dark adaptation and reduced visual acuity (4, 5).

The current gold standard methods for identifying retinal neural dysfunction and damage are multi-focal electroretinogram (mfERG) and frequency domain optical coherence tomography (FD-OCT). Studies have shown altered mfERG responses (6, 7), retinal nerve fibre layer (RNFL) thinning (8, 9) and a loss of central visual field sensitivity (4, 8, 10) in patients without retinopathy (11). Another study using OCT imaging of the retina reported a high correlation between perimetric function loss and retinal neuropathy in patients with no or mild DR (12). Thus, functional testing can help understand patients’ symptoms prior to DR and may be used to counsel patients about the importance of optimizing metabolic control. It is interesting that the latest position statement by the American Diabetes Association defined diabetic retinopathy as neurovascular complications rather than microvascular complications of both type 1 and type 2 diabetes (13), and the term ‘diabetic retinopathy’ has now been replaced by ‘diabetic retinal disease’ (14).

Over the past decade, a growing body of evidence has demonstrated corneal nerve fiber loss in diabetic patients through the application of corneal confocal microscopy (CCM). CCM allows quantification of corneal c-nerve fibers, enabling early diagnosis and stratification of the severity of diabetic peripheral neuropathy (DPN) (15, 16). Several cross-sectional studies have evaluated the ability of CCM to diagnose clinical levels of DPN in comparison to a range of other diagnostic tests (17) and a stepwise deterioration in corneal nerve morphology in patients with pre-proliferative and proliferative DR has been demonstrated (18–21).

Whilst there is a recognised association between the main microvascular complications, this has only been investigated in advanced cases. Indeed, the Rochester Diabetic Neuropathy Study has shown that markers of micro vessel damage such as DR and proteinuria or microalbuminuria (MA) are the strongest predictors for the severity of DPN (22). Furthermore, a strong association has been observed between cardiac autonomic neuropathy (CAN) and proliferative DR in patients with type 1 diabetes mellitis (T1DM), suggesting an underlying etiologic link (23). Although the underlying molecular mechanisms linking DR and cardiovascular disease are still under debate, similarities in their pathophysiology have been notable (24). To our knowledge, only one published study has investigated the temporal relationship between ocular neural and vascular damage. Their results suggested progressive loss in neuroretinal layer thickness in people with no or minimal DR after four-year follow-up, independent of DR or progression of DR (25). However, that study did not assess corneal nerves. Both CCM and OCT imaging demonstrate potential for the clinical application of monitoring diabetic retinal disease alongside functional assessment. The present study aimed to evaluate functional and structural changes in the retinal nerve layer and corneal c-fibres in patients with T1DM without clinical evidence of retinopathy at the time of examination

## Materials and methods

### Patient population

This was a preliminary observational, retrospective study. Twenty patients with T1DM (Age: 47.0 ± 2.5 years) and 15 age-matched healthy control subjects (Age: 45.0 ± 4.0 years) underwent medical, neurological and ophthalmic examination, which included structural and functional assessment of the retinal and corneal nerves. All subjects underwent detailed ophthalmic assessments, including slit-lamp biomicroscopy, Spectral Domain Optical Coherence Tomography (SD-OCT), visual field assessment with using Standard Automated Perimetry (SAP), Flicker Defined Form High Edge Perimetry (FDF), Corneal Confocal Microscopy (CCM) and Non-contact corneal Aesthesiometry (NCCA). Inclusion criteria were having type 1 diabetes (T1DM) with no evidence of diabetic retinopathy (DR) or other causes of neuropathy.

Exclusion criteria were any other cause of ocular or peripheral neuropathy other than diabetes, active corneal disease or surgery, wearing hard or RGP contact lenses and a history of laser treatment for DR. This research adhered to the tenets of the declaration of Helsinki. Written informed consent was obtained from all subjects prior to participation. None of the subjects suffered from other causes of neurodegenerative conditions (e.g. Parkinson’s disease, multiple sclerosis, Huntington’s disease) and no retinal conditions that can affect RNFL (e.g. Glaucoma)

### Retinal assessment

#### Fundus photography

The grade of retinopathy of each patient was obtained from the UK National Screening programme based on the “Early Treatment of Diabetic Retinopathy Study” (EDTRS) grading system (26), and only patients without a current or past history of DR were included. To rule out retinopathy in control subjects, a standard field fundus photograph was captured using a Canon CR-2 Plus digital non-mydriatic retinal camera (Canon Healthcare Technologies, Melville, New York, USA) and the image was assessed by a trained, study certified optometrist.

#### Spectral-domain optical coherence tomography (retinal structure)

Retinal nerve fiber layer (RNFL) thickness was assessed using SD-OCT with the Spectralis HRA+OCT, Heidelberg Engineering, Germany. The Spectralis OCT uses a dual-beam SD-OCT and a confocal laser scanning ophthalmoscope (CSLO) that works by emitting a super luminescent diode light with a center wavelength of 870nm and an infrared scan to simultaneously provide images of ocular microstructures. The instrument uses 1024 A-scan points from a 3.45mm circle centered on the optic disc, which the examiner manually centers. The imaging included a 97-line raster volume scan, 20 × 20 degrees centered on the optic nerve; a 49-line raster volume scan, 20 × 20 degrees centered on the fovea, and a standard 12-degree circular scan centered on the optic nerve. Scans were acquired with eye-tracking, and averaging was set at 16 frames for raster scans and 40 frames for circular scans. The instrument requires scan averaging to reduce noise, thus increasing the effective acquisition time. Scans were repeated if image overlap was noted during averaging or if the image quality was less than 25 dB. RNFL measures were reported for global RNFL thickness, as well as 6 values representing sections of the optic nerve: nasal (N), superior nasal (SN), superior temporal (ST), temporal (T), inferior temporal (IT) and inferior nasal (IN).

#### High edge perimetry (retinal function)

The function of the retinal ganglion layer was assessed with FDF using Heidelberg High Edge Perimetry (HEP), in addition to SAP. FDF Perimetry targets primarily the magnocellular signal pathway in which the retinal ganglion cells are sparsely distributed in the retina. In contrast, standard white-on-white perimetry targets all retinal ganglion cells where other cells may take over M-cell function and a visual field defect might be masked. The exact retinal cellular mechanisms of FDF still remain unclear; however, evidence suggests that FDF perimetry could be more sensitive than SAP and other types of perimetry for detecting early functional loss (27). Thus, we included FDF in addition to SAP to evaluate its structure-function relationship with retinal and corneal nerves in patients with T1DM. We were able to use combined Spectralis RNFL and HEP visual field assessments, which enables concurrent assessment of damage to the optic nerve head (ONH), retinal ganglion cell (RGC) loss and RNFL thickness; a novel aspect of this study.

### Corneal assessment

#### Corneal confocal microscopy (corneal structure)

All subjects underwent examination with the Heidelberg Retina Tomograph (HRT III) in vivo corneal confocal microscope (CCM) based on our established protocol (28). Six images per subject from the center of the cornea were selected and examined in a masked and randomised fashion. Three corneal nerve parameters were quantified: (i) corneal nerve fiber density (CNFD) – the total number of major nerves/mm2 of corneal tissue; corneal nerve branch density (CNBD) – the number of branches emanating from all major nerve trunks/mm2 of corneal tissue and (iii) corneal nerve fiber length (CNFL) – the total length of all nerve fibers and branches (mm/mm2) within the area of corneal tissue.

#### Corneal aesthesiometry (corneal function)

Corneal sensitivity was quantified using a non-contact corneal aesthesiometer (NCCA) (Glasgow, Caledonian University, UK) which uses a puff of air through a bore of 0.5mm in diameter and lasting 0.9 seconds to exert a force expressed in millibars (mbars) (29).

### Neuropathy assessments

All patients and control subjects underwent a detailed evaluation of neurological symptoms using the Neuropathy Symptom Profile (NSP) (30, 31). Neurological deficits were assessed using the neuropathy disability score (NDS), which includes evaluation of vibration, pin-prick and temperature perception, as well as the presence or absence of ankle reflexes, to establish the severity of neuropathy (31). Quantitative sensory testing included warm sensation and heat induced pain (WS, HIP) to assess C fibers and cold sensation and cold induced pain (CS, CIP) to assess Að fibers, using the MEDOC *TSA II* (Medoc Ltd., Ramat Yishai 30095, Israel) device on the dorsum of the left foot. Sensory thresholds were determined by the method of limits.

### Statistical analysis

SPSS 20.05.0 for Windows was used to compute the results. Analysis included descriptive and frequency statistics. All data are expressed as mean ± SEM. One-way analysis of variance (ANOVA) with Scheffe *Post-hoc* tests were used to study differences between means. The Pearson test was used to analyze correlations between potentially related variables.

## Results

The clinical and demographic results of all participants are presented in **Table 1**. None of the patients had clinical evidence of DR or nephropathy and only mild neuropathy based on the NDS score (score of less than 3).

**Table 1:** Demographic data and neurological assessments.

	Healthy Control Subjects (HCS)	Patients (T1DM)	P-value
No (F/M)	15	20	NS
Age (yrs)	45.1±4.4	47±2.5	NS
Duration Diabetes (yrs)	0	27.22±3.27	NS
HbA1c (%)	5.5±0.1	7.33±0.64	<0.004
HbA1c (mmol/mol)	36.24±1.20	56.68±7.02	<0.004
Retinopathy (%)	0	0	NS
eGFR (ml/min/l)	87.33±1.78	74.81±5.71	NS
ACR (mg/mmol)	0.67±0.17	0.90±0.47	NS
NDS (0-10)	0	2.0 ±0.5	<0.0001
NSP (0-38)	0	3.06±1.50	<0.0001
VPT (v)	6.58±1.79	9.89±1.84	0.03
CS (^o^C)	28.31±1.16	24.81±1.68	0.02
WS (^o^C)	35.99±0.75	39.32±0.77	0.004

Data presented mean± SE. HbA1c, Glycated haemoglobin; eGFR, Estimated glomerular filtration rate; ACR, Albumin to creatine ratio; NDS, Neuropathy disability score; NSP, Neuropathy symptoms profile; VPT, Vibration perception threshold; CS, Cold sensation; NS, Not significant; WS, Warm Sensation.

### Ophthalmic results

#### Retina

**Figure 1:**
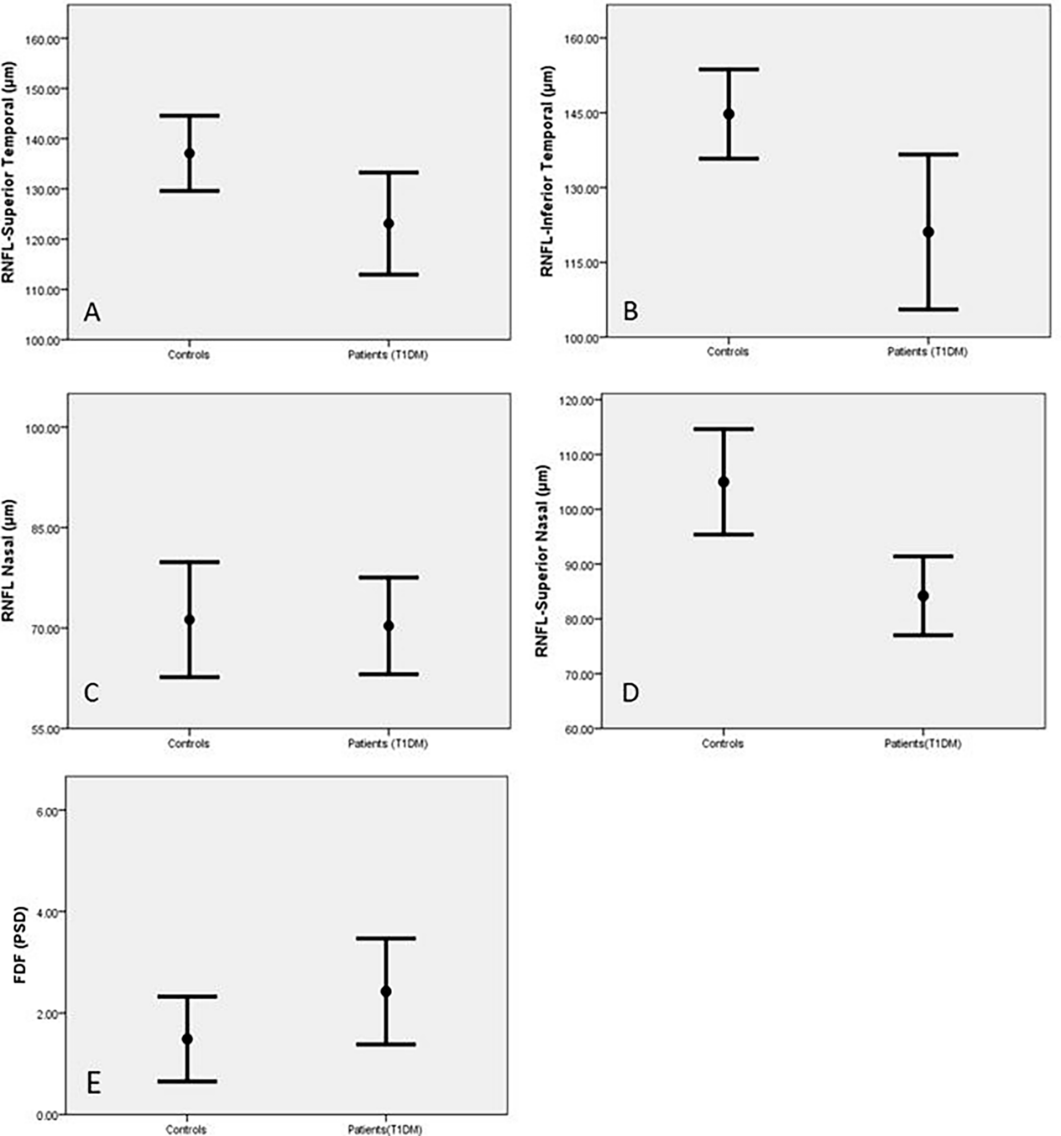
Retinal Nerve Fibre Length (RNFL) in the superior temporal **(A)**, inferior temporal **(B)**, inferior nasal **(C)** and superior nasal **(D)** areas and FDF **(E)** in control subjects and patients with T1DM expressed as Mean and SEM.

RNFL thickness was significantly reduced in the superior nasal (SN) (p=0.001) superior temporal (ST) (p=0.02) and inferior temporal (IT) (p=0.004) sectors of the optic nerve head in diabetic patients compared to control subjects (**Table 2** and **Figure 1**). There was no significant reduction in global RNFL (p=0.093), or for nasal (N)(p=0.641), inferior nasal (IN)(p=0.722) or temporal (T) (0.117) ONH sectors. Visual field assessment did not differ between diabetic patients and control subjects for FDF perimetry (p=0.141) or SAP perimetry (p=0.188) (**Table 2**).

**Table 2:** Corneal and retinal nerve fiber layer functional & structural assessments.

	Healthy Control Subjects (HCS)	T1DM	P Value
**Corneal Sensitivity (Corneal Function)**			
NCCA (mbar)	0.48±0.1	1.1±0.13	0.001
**Corneal Confocal Microscopy (Corneal Structure)**			
CNFD (no/mm^2^)	34.91±1.64	27.82±1.79	0.01
CNBD (no/mm^2^)	78.60±5.93	52.79±6.16	0.006
CNFL (mm/mm^2^)	24.84±1.44	20.25±1.10	0.02
**Visual Field Perimetry ( Retinal Function)**			
HEP: FDF (PSD)	1.48 ± 0.37	2.42 ± 0.49	0.141
HEP: SAP (PSD)	1.45 ± 0.30	2.09 ± 0.43	0.188
**Retinal Nerve Fiber Layer Thickness (Retinal Structure)**			
RNFL- Global (µm)	96.50±2.2	90.42±2.71	0.093
RNFL- Nasal (µm)	71.25±3.90	70.31±3.43	0.641
RNFL- Inferior Nasal (µm)	100.75±5.28	100.31±4.79	0.722
**RNFL- Inferior Temporal (µm)**	144.7±4.06	121.10±7.39	0.004
RNFL- Temporal (µm)	77.00±4.48	69.00±2.43	0.117
**RNFL- Superior Temporal (µm)**	137.08±3.40	123.1±4.83	0.02
**RNFL- Superior Nasal (µm)**	105.00±4.37	84.21 ±3.42	0.001

NCCA, Non-contact Corneal Aesthesiometry; CNBD, Corneal Nerve Branch Density; CNFD, Corneal Nerve Fiber Density; CNFL, Corneal Nerve Fiber Length; HEP, High Edge Perimetry; FDF, Flicker Defined Form; PSD, Pattern Standard Deviation; SAP, Standard Automated Perimetry; RNFL, Retinal Nerve Fiber Layer; NS, Non-significant p-value.

For retinal assessments, the Heidelberg software for both OCT and HEP indicated abnormal results based on the age-matched normative values of the designed systems. Based on this, RNFL was abnormal in 32% of patients, FDF was abnormal in 61% and standard white perimetry was normal for all cases.

#### Cornea

**Figure 2:**
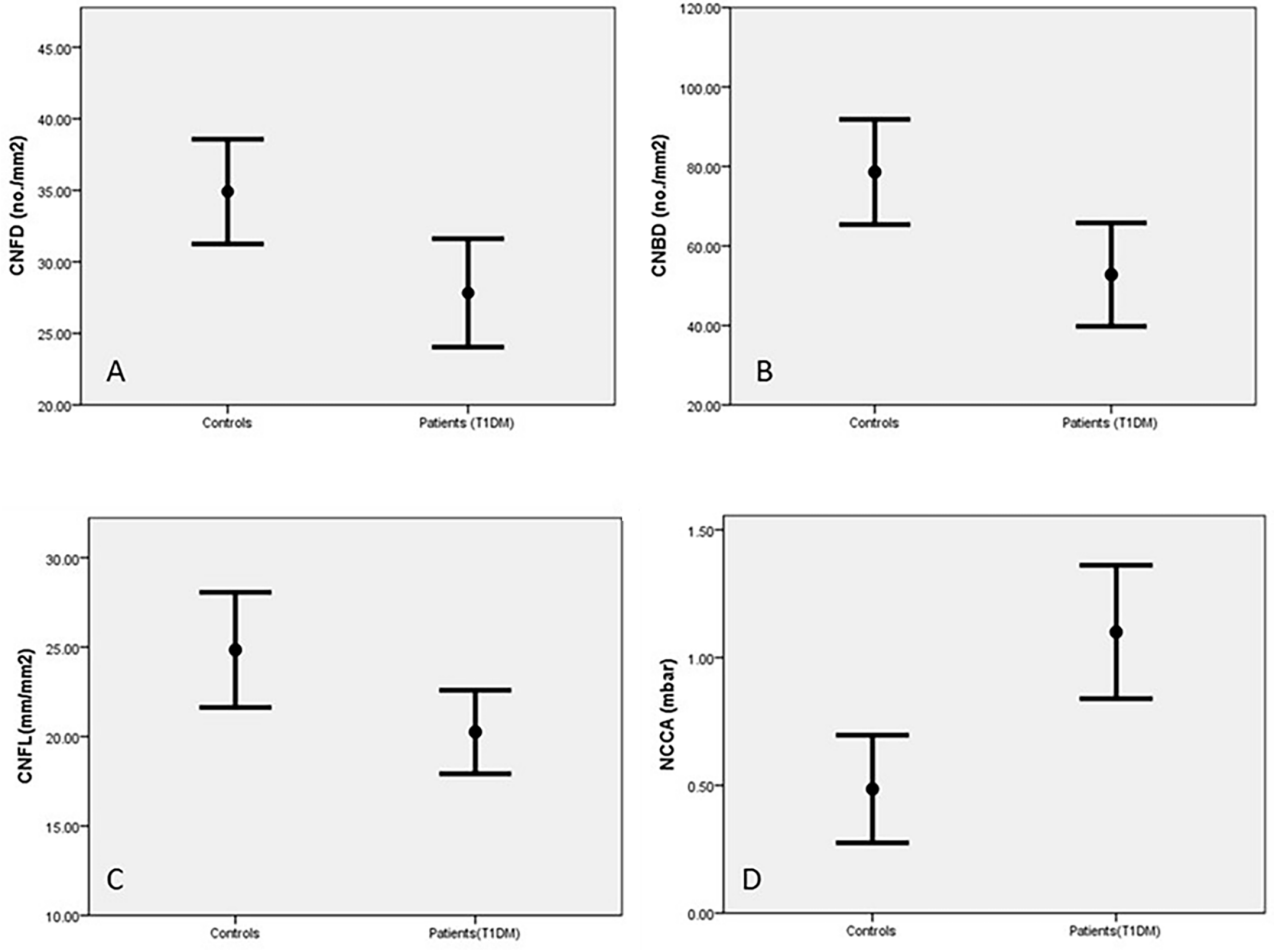
Corneal nerves parameters in control subjects and patients with T1DM corneal nerve fiber density (CNFD) **(A)**, corneal nerve branch density (CNBD) **(B)**, corneal nerve fiber length (CNFL) **(C)** and Non-contact corneal Aesthesiometry (NCCA) **(D)** (results are expressed as Mean and SEM).

Corneal sensitivity was significantly reduced in diabetic patients compared to control subjects (p=0.001, **Table 2**). CNFD (p=0.01), CNBD (p=0.006) and CNFL (p=0.01) were also significantly reduced in diabetic patients compared to the control group (**Table 2** and **Figure 2**). Abnormalities in corneal measurements were defined as a value, which was more than 2SD from the value found in control subjects. Based on this definition, corneal sensitivity was abnormal in 47% of patients, whilst CNFD was abnormal in 27.8%, CNBD at 23.4% and CNFL in 16.7%.

### Correlations

There was a significant correlation between the duration of diabetes and RNFL thickness (IT, ST, SN), CNFL and NCCA, as well as FDF. HbA1c correlated significantly with RNFL (IT) and CNFL (**Table 3**). There was no correlation between corneal and retinal markers of neural damage.

**Table 3:** Correlation between duration of diabetes & HbA1c with corneal and retinal markers of neural damage.

		RNFL (IT)		RNFL (ST)	RNFL (SN)	CNFD	CNFL	NCCA	FDF (PSD)	SAP (PSD)
	Duration of Diabetes	r P	-.584 .001	-.418 .019	-.558 .001	-0.25 0.3	-.402 .031	.531 .002	.406 .029	.366 .103
	HbA1c	r P	-.380 .050	-.054 .788	-.318 .106	-0.091 0.7	-.417 .030	.315 .109	.189 .367	.018 .940

## Discussion

Diabetic microvascular complications result in considerable morbidity with both microalbuminuria and the severity of DR relating to the severity of DPN (22). The temporal relationship for the development of microvascular complications has been systematically assessed in very few studies (32, 33). Recent studies have shown that hyperglycemia can affect not only the retinal vasculature but also the neuro-retina, resulting in a loss of visual acuity (4). Furthermore, studies have also shown altered multi-focal electroretinogram (mfERG) responses in patients with diabetes (6).

It is established that during diabetes, retinal glial, neural and microvascular dysfunction is interdependent and essential for the development of DR (3). Neuronal degeneration and early retinal dysfunction have been demonstrated in various animal models of diabetes (34–36). Furthermore, postmortem studies have revealed neuronal degeneration and apoptosis in the ganglion cell layer (37). Spectral-domain OCT (38) has shown a thinning of the RNFL (39, 40) and photoreceptor (41) layers in diabetic patients without retinopathy. Further thinning of the ganglion cell layer and inner plexiform layers have also been reported in patients with mild (42) and advanced DR (9). Hence, neurodegeneration may play a key role in the development and progression of DR, and structural RNFL monitoring may help to identify diabetic patients who would benefit from neuroprotection (11). Our findings demonstrated no significant global RNFL thinning in patients with diabetes when compared to healthy controls. However, significant thinning was evident in the ST, SN and IT peripapillary sectors. Sectoral thinning has been reported previously in T1DM, prior to retinopathy, with conflicting reports of the inferior (43) and superior (44) quadrant thinning occurring first. The pathophysiology of non-uniform peripapillary thinning is not fully understood. One possible explanation for thinning of the superior and inferior quadrants prior to the temporal and nasal may be due to relative RNFL sectoral thicknesses. The thicker superior and inferior quadrants may have a higher sensitivity to oxygen deprivation due to their higher metabolic demand, thus being affected more by the metabolic stress from the disease. Of course, these findings would require further investigation.

Additionally, we found significant correlations of peripapillary sectoral thinning with HbA1c and duration of diabetes. HbA1c levels are well known to be linked to microvascular abnormalities, and intensive glycaemic control therapy reduces the risk of any retinopathy developing (45). It may be that neuro-retinal damage is more sensitive to HbA1c levels in patients with T1DM, thus associating with neuro-retinal thinning prior to DR in our study. However, we recognise that glycaemic control fluctuates in many patients, and our cross-sectional study may not have captured participants’ average HbA1c level. This may explain why our findings contradict those of a previous longitudinal study in patients with T1DM, in which no relationship between progressive thinning and consistent HbA1c levels were found over a 4 year period (25).

Research has shown that several pathways are likely involved in neuronal cell death in the retina of patients with diabetes (46). Animal models and studies of intraocular fluid in eyes with diabetes have suggested elements of oxidative stress, glutamate excitotoxicity and hyperglycemia-related production of advanced glycation end-products (AGEs) as potential molecular mechanisms underlying neuro-retinal loss (47). There has been recent interest in the specific role of Müller cells in diabetic neurodegeneration. Müller glia provide critical homeostatic and trophic support for the neuronal layers and vasculature of the retina and hyperglycemia may target these cells, thus inducing neuroinflammation and leading to ganglion cell loss. A recent study by Miller and colleagues (48) assessing diabetic mice, suggested that enhancement of the protein regulated in development and DNA damage responses 1 (REDD1) expression leads to a failure of Müller cells to properly respond to the diabetic metabolic environment.

Visual field sensitivity and visual function have also demonstrated reductions in patients with diabetes (4, 8, 12, 49) prior to, or with minimal retinopathy. In the current study, we found no significant difference between the patient population and healthy controls when assessing both types of perimetric results, thus our results do not support these previous findings (42). However, in comparison to van Dijk and colleagues, we used different methods of perimetry. We also only included patients with no DR, whereas that study included patients with minimal DR. It may be that our participants had milder neurovascular changes in comparison, thus demonstrated structural changes prior to detectable functional loss.

Previous studies have also shown a stepwise deterioration in corneal nerve morphology in patients with pre-proliferative and proliferative DR (18–20), and more recently corneal nerve damage has also been detected in patients without retinopathy (18, 20, 21, 50). Our results support these previous findings with significant reductions in all three corneal nerve parameters in patients with T1DM compared to healthy controls, as well as a significant reduction in corneal sensitivity. These findings highlight the potential use of CCM imaging for detecting early neurovascular changes in patients with diabetes. Furthermore, CNFL demonstrated a significant relationship with duration of diabetes and HbA1c.

Although we found reductions in both the RNFL and corneal nerve parameters independently, we and others (51) found no correlation between neural changes in the retina (central nervous system) and cornea (peripheral nervous system). At present, the reason for this finding is unclear. It may be that corneal and retinal neurodegeneration occur as independent pathological processes. Regions of retinal non-perfusion have previously demonstrated profound inner-retinal thinning (52) and it may be that retinal microvascular changes are linked to retinal thinning but not corneal nerve degeneration. However, the argument over which causes the other within the retina is still under debate and yet to be determined (5).

Additionally, there is evidence for defective direct insulin signalling and loss of insulin-derived neurotrophic support possibly contributing to peripheral neuropathy in patients with type 1 diabetes (53). One study reported a rapid improvement of epidermal innervation in mice paws, through doses of insulin insufficient to alter glycemia (54). Another study which applied daily insulin to the eyes of both control and streptozotocin-diabetic mice without altering blood glucose levels found that it successfully prevented depletion of nerves of the sub-basal plexus (55). This evidence suggests that the neuroprotective role of insulin can be independent of effects on hyperglycemia and local manipulation of insulin signaling may offer novel therapeutic peripheral nerve repair.

Based on our definition, 16.7-27.8% of patients were categorized as ‘abnormal’ using CCM, depending on the nerve parameter used for definition, and 32% based on RNFL thickness alone. These values suggest that several subjects who do not meet the referral criteria into the hospital eye service (HES) based on retinopathy may require closer monitoring based on ocular nerve changes. The future approach for developing new pharmacological agents for DR and other microvascular complications of diabetes could also use a combined approach for targeting neurodegeneration, inflammation, and oxidative stress (56), focusing on the potential of novel therapies for neuroprotection (57).

The findings of this preliminary study may not only apply to diabetic neuropathy but also other to systemic neurological conditions. Subjects with diabetes are known to have a higher prevalence of dementia and other neurodegenerative diseases, such as Parkinson’s disease in comparison to age-matched controls (58). Additionally, a number of pathways that are involved in these neurodegenerative diseases have been found to be altered in the retinae of patients with diabetes (59). A recent study found that testing patients with T2DM using microperimetry could identify a risk of developing Alzheimer’s disease, with retinal sensitivity relating to cognitive status (60). Thus, there may also be scope for the application of neuro-retinal imaging and corneal nerve imaging to identify patients with diabetes who are at risk of this development, due to the relationship with brain neurodegeneration.

Although we used a different type of perimetry measurement, in our study we found no significant changes in visual field results, despite RNFL thinning and corneal nerve changes. Thus, structural changes may precede functional loss in these patients, and nerve imaging detected abnormalities prior to functional assessment. Furthermore, objective measures would arguably be more sensitive and reliable than subjective tests such as perimetry.

In the present study, we demonstrate that novel *in-vivo* ophthalmic techniques can identify corneal and retinal neurodegeneration in patients with T1DM without clinical evidence of retinopathy. One may argue that the very fact that the patients in our study have no evidence of retinopathy, despite a long duration of diabetes, suggests that they are less prone to retinopathy and indeed the other complications. However, the demonstration of neuronal dysfunction and damage in the retina and cornea would argue against this and suggest that neuronal damage may precede vascular damage in the eye. Indeed, our study has some limitations including long duration of the disease and a small sample size. Future, prospective studies are needed to establish the exact temporal relationship between the development of retinopathy and neuropathy. This will allow to development of better targeted and more efficient therapeutic approach in the early stages of the disease.

## Data availability statement

The raw data supporting the conclusions of this article will be made available by the authors, without undue reservation.

## Ethics statement

This research adhered to the tenets of the Declaration of Helsinki and was approved by the Manchester Research Ethics Committee and the ethics review board of the university of Manchester. The participants provided their written informed consent to participate in this study. The data analyzed in this study were originally collected at the University of Manchester between 2012 and 2015 under approval from the Manchester Research Ethics Committee and the University of Manchester Research Ethics Review Board. Ethical review and approval was not required for the present study in accordance with local legislation and institutional requirements because this study represents a secondary analysis of data previously collected with ethical approval. Written informed consent for participation was obtained from all participants at the time of original data collection.

## Author contributions

MT designed the study, researched and analyzed the data and wrote the paper. JC, HF, MT, contributed to writing and analyzing the data. All authors contributed to the article and approved the submitted version.

## Funding

This work was supported by Heidelberg Engineering UK.

## Acknowledgments

Support from Prof. Andrew JM Boulton and Prof. Rayaz Malik is acknowledged for the initial study. Dr Maryam Ferdousi and Dr Ioannis Petropoulos undertook corneal confocal microscopy in a proportion of the study patients.

## Conflict of interest

The authors declare that the research was conducted in the absence of any commercial or financial relationships that could be construed as a potential conflict of interest.

## Correction note

A correction has been made to this article. Details can be found at: 10.3389/fendo.2026.1809367.

## Publisher’s note

All claims expressed in this article are solely those of the authors and do not necessarily represent those of their affiliated organizations, or those of the publisher, the editors and the reviewers. Any product that may be evaluated in this article, or claim that may be made by its manufacturer, is not guaranteed or endorsed by the publisher.

## Glossary

**Table:**

ACR	Albumin to Creatinine Ratio
ANOVA	Analysis of Variance
CAN	Cardiac Autonomic Neuropathy
CIP	Cold Induced Pain
CCM	Corneal Confocal Microscopy
CNBD	Corneal Nerve Branch Density
CNFD	Corneal Nerve Fiber Density
CNFL	Corneal Nerve Fiber Length
CS	Cold Sensation
CSLO	Confocal Laser Scanning Ophthalmoscope
dB	Decibels
DR	Diabetic Retinopathy
DM	Diabetes Mellitus
EDB	Extensor Digitorm Brevis
eGFR	Estimated Glomerular Filtration Rate
ETDRS	Early Treatment of Diabetic Retinopathy Study
FDF	Flicker Defined Form
FD-OCT	Frequency Domain OCT
HbA1c	Glycated Hemoglobin
HEP	High Edge Perimetry
HIP	Heat Induced Pain
HRT	Heidelberg Retina Tomograph
IN	Inferior Nasal
IRSLO	Infrared Scanning Laser Ophthalmoscope
IT	Inferior Temporal
MA	Micro Albuminuria
mfERG	Multi-focal Electroretinogram
NCCA	Non-contact Corneal Aesthesiometry
NDS	Neuropathy Disability Score
NSP	Neuropathy Symptoms Profile
OCT	Optical Coherence Tomography
PDR	Proliferative Diabetic Retinopathy
PSD	Pattern Standard Deviation
RGC	Retinal Ganglion Cell
RNFL	Retinal Nerve Fiber Layer
SAP	Standard Automated Perimetry
SD-OCT	Spectral Domain Optical Coherence Tomography
SE	Standard Error
SN	Superior Nasal
ST	Superior Temporal
T1DM	Type 1 Diabetes Mellitus
T2DM	Type 2 Diabetes Mellitus
VPT	Vibration Perception Threshold
WS	Warm Sensation
