# Correction: Neurodegeneration of the cornea and retina in patients with type 1 diabetes without clinical evidence of diabetic retinopathy

**DOI:** 10.3389/fendo.2026.1809367

**Published:** 2026-03-06

**Authors:** Josie Carmichael, Hassan Fadavi, Mitra Tavakoli

**Affiliations:** 1 Exeter Centre of Excellence for Diabetes Research, National Institute for Health and Care Research (NIHR) Exeter Clinical Research Facility, University of Exeter Medical School, Exeter, United Kingdom; 2 Peripheral Neuropathy Group, Imperial College, London, United Kingdom

**Keywords:** neurodegenaration, type 1 diabetes mellitus, retinopathy, neuropathy, corneal confocal microscopy, optical coherance tomography

The **Ethics statement** was erroneously given as “Ethical review and approval was not required for the study on human participants in accordance with the local legislation and institutional requirements. The patients/participants provided their written informed consent to participate in this study.” The correct **Ethics statement** appears below.

“This research adhered to the tenets of the Declaration of Helsinki and was approved by the Manchester Research Ethics Committee and the ethics review board of the university of Manchester. The participants provided their written informed consent to participate in this study. The data analysed in this study were originally collected at the University of Manchester between 2012 and 2015 under approval from the Manchester Research Ethics Committee and the University of Manchester Research Ethics Review Board. Ethical review and approval was not required for the present study in accordance with local legislation and institutional requirements because this study represents a secondary analysis of data previously collected with ethical approval. Written informed consent for participation was obtained from all participants at the time of original data collection.”

The original version of this article has been updated.

